# Editorial: Patient-derived tumor models for drug development

**DOI:** 10.3389/fonc.2023.1243534

**Published:** 2023-07-07

**Authors:** Hatim E. Sabaawy, Massimo Broggini, Shiv K. Gupta

**Affiliations:** ^1^ Department of Medicine, Division of Medical Oncology, University of Colorado Anschutz Medical Campus, Aurora, CO, United States; ^2^ Laboratory of Molecular Pharmacology, Department of Oncology, Istituto di Ricerche farmacologiche Mario Negri (IRCCS), Milan, Italy; ^3^ Department of Radiation Oncology, Mayo Clinic, Rochester, MN, United States

**Keywords:** patient-derived models of cancer, organoid, patient-derived xenograft (PDX), syngeneic graft, pre-clinical drug screen for cancer, immune checkpoint inhibitors, precision oncology, precision medicine

The need for pre-clinical models to better predict the individual clinical outcome has driven the development of improved patient-derived models. Among variety of cancer models, patient derived xenografts (PDXs) and patient-derived organoids (PDOs) have shown promise to advance the field towards realizing the goals of precision cancer medicine (1–4). However, despite tremendous progress in preclinical models and technological advancement in drug screening, tumor models are not yet perfect. Ongoing studies continue to improve these models especially to address the need to - 1) reflect the intra- and inter-tumoral heterogeneity, which include but not limited to key molecular determinants of prognosis and therapeutic response (5); 2) unmask the effects of the tumor microenvironment (TME) which supports tumor progression and recurrence (6), and 3) allow testing of broader panels of novel drugs and targeted combinations, along with limiting exposure to unforeseen toxicity. These recent advances in tumor models have increasingly been incorporating components of the human immune system and have shown great potential for guiding personalized cancer therapies.

Through the collection of several insightful reviews, perspectives, and original research articles, this Research Topic aims to highlight recent progress and facilitate scintillating discussion to advance the preclinical research aligned with the goals of precision cancer medicine. We summarize here some of the key aspects covered in the various contributions, offering the readers a comprehensive understanding of this impactful area of research.

Cancer cell line models, for decades, have been extensively used in studies to gain valuable insights into tumor progression and drug responses. However, due to clonal selection and repeated passages under serum-containing medium for years, cell lines tend to have genetic drift from the original tumors (7). Nevertheless, cancer cell lines remain a primary resource to study molecular mechanisms and drug responses. Therefore, establishing cancer cell lines, especially for the rare tumor entities with limited tissue availability, is highly important. Andus et al., report the establishment and characterization of three patient derived cell lines (PDCs) for non-small cell lung cancer (NSCLC) subtypes: adeno-, squamous cell, and pleomorphic carcinoma. PDCs of pleomorphic cell type carcinoma, for which the biological and drug response information are scarce, represent new preclinical models. Similarly, Driesch et al., report the establishment of a PDC as well as the first in-depth characterization of Medullary pancreatic carcinoma, a rare subtype of pancreatic ductal adenocarcinoma. These PDC models, with the detailed characterization of their molecular and morphological features and drug-sensitivity profile, represent valuable pre-clinical tools for advancement of precision cancer therapy.

Models of prostate cancer (PCa) are also scarce, with nearly a dozen established cell lines and fewer organoid models (8), yet personalized approaches for PCa therapy have been promising (9). Developing additional models to represent heterogeneity of PCa, that include adenocarcinoma and neuroendocrine phenotypes, are necessary. Beraud et al., report the development and characterization of five PDX models and their utility in evaluating therapeutic modalities such as androgen deprivation, PARP inhibitors and chemotherapy. These prostate cancer PDXs included hormone naïve, castrate resistant and neuroendocrine subtypes, and revealed a favorable response to PARP inhibitors in a *BRCA2* mutant neuroendocrine model. Similarly, Yu et al. report the generation of a large panel of 171 PDX models, including 85 colorectal cancer (CRC), 21 esophageal, and 65 gastric cancer PDX models. The authors correlated the clinicopathological and molecular features of tumors with PDX take rates and demonstrated that tumor biomarkers and proliferation index could be validated in PDXs. Interestingly, cetuximab had a significantly lower overall tumor response rate in patients with RAS mutation. Screening of drug sensitivity in PDX models led the authors to identify two patients with K-Ras mutant tumors that responded to cetuximab, demonstrating the use of PDX models in patient case reports of CRC therapy. Two different incisive reviews by Liu et al. and Aaltonen et al., describe recent advances and knowledge gaps in lung cancer and neuroblastoma research, respectively, where patient derived models have been extensively used in co-clinical avatar studies for precision medicine.

Beyond the evaluation of drug distribution and therapeutic responses in live animals, PDX models can serve in multiple ways. For example, readily available PDX samples can be leveraged to identify novel targets and biomarkers through exploratory studies, which otherwise may not be feasible due to paucity of patient tissue. Ferrarini et al., performed a proof-of-concept study using lung adenocarcinoma PDXs and demonstrated plurimetabolic state in 9 of 14 PDXs analyzed. Expanding on the utility of PDXs in Neurooncology, Alcaniz et al., established and characterized 23 IDH-wt glioblastoma (GBM) PDXs, initially as heterotopic GBM PDXs, then propagated as brain orthotopic models, to assess drug resistance, and the impact of TME and blood brain barrier (BBB), which are key limitations to therapeutic response in orthotopic GBM models (10). Deng et al., demonstrated the efficacy of small molecule inhibitor of Sonic hedgehog (Shh) signaling in limiting cholesterol metabolism which supports medulloblastoma growth. Despite the extensive utility of PDXs propagated in immune compromised animals and ex vivo in tissue culture, PDX models have limited applicability specially to study TME or tumor immune response. Furthermore, tumor take rates are variable, consequently only a fraction of any given tumor type can be timely established as PDXs for co-clinical avatar studies. Slower growth rate and clonal selection in the mouse background can also cause unpredictability, these challenges have been overlooked with their growing list of advantages in oncology.

Patient-derived tumor organoids (PDOs) are cultures of cells resected tumors from an individual patient. PDOs can serve as models to understand patient-specific drug responses and to investigate cancer cell growth, and molecular analysis. The overall importance of PDOs has been nicely highlighted by a review article (Verduin et al.) and a perspective article (Wang et al.). The review collected data obtained from 60 published data spanning different tumor types. The authors concluded that PDOs have indeed the potential to predict response to treatment and have high translatability to the clinics. They highlighted the possibility to use PDOs in high-throughput studies to define targets, examine treatment response and further personalize these treatments. The authors especially highlight the discordance between *in vitro* vs. *in vivo* drug efficacy, that could be due to dose limiting toxicity in normal tissues. Therefore, evaluating drug sensitivity using normal tissue organoids in parallel with that in cancer organoids is a valuable addition to the organoid based co-clinical avatars in precision cancer medicine. Wang et al. specifically touched on the potential of PDOs as models in cancer immunotherapy. The use of complex organoids with enhanced heterogeneity of cell populations is an approach we must encourage to have the potential of studying the interaction between cancer cells and stroma, especially immune cells, at the basic and translational levels. Since PDOs lack vasculature, nervous system and drug metabolic pathways, this platform cannot be used for testing all drugs. Considering these limitations, Tan et al., adapted a strategy to use PDOs with available PDXs, established for the advanced gallbladder cancer, for initial rapid drug screening and *in vivo* drug efficacy validation, respectively, and allude to making these two models complementary. This approach can guide the individualized treatment strategies for cancer patients, when PDXs could be established in timely fashion.

Cancer immunotherapy has revolutionized the field of oncology by prolonging survival and quality of life for cancer patients. However, a comprehensive *in vivo* evaluation of immunotherapies is not possible in PDX models raised in immune compromised animals. Humanized mouse models, i.e., severely immune deficient strain engrafted with a human immune system, represent the most relevant platform to test and validate cancer immunotherapy approaches. Here, two original manuscripts describe the results from *in vivo* evaluation of immune checkpoint inhibitors (ICIs) using humanized mouse models. First, Tan et al, report that gallbladder tumor PDXs in humanized mice, generated with human peripheral blood mononuclear cells and treated with nivolumab, show immune cell death in tumors and that PD-L1 expression is not a direct indicator of the tumor suppressive activity of ICI, a finding that reflects the consensus on the unreliability of PD-L1 expression as a biomarker and the need for better biomarkers for response to ICI-based immunotherapy. Second, Marie et al., report the improvement of the traditional CRC PDX models by developing novel humanized CRC PDX models utilizing autologous (from same CRC patient) or allogeneic (from healthy donor) PBMCs. Marie et al., elegantly demonstrated that while immune (HLA)-mismatched allogeneic models developed graft-vs-tumor effects limiting their utility, autologous humanized mice were proven useful, when used within a window-of-treatment, in detecting T-cell responses, myeloid cell infiltration and predicting responses to ICIs and potential combination therapies. The authors also demonstrate that modulation of immuno-suppressive cells, such as regulatory T cells within the TME can augment the efficacy of ICI agents in CRC. The use of newly available mouse strains with expression of human cytokines or those lacking mouse major histocompatibility proteins could potentially improve the support of engrafted immune cells and the therapeutic window in humanized animals.

Overall, the collection of articles in this Research Topic provides important additions to the arena of clinically relevant patient derived models to be utilized for drug testing and offers a roadmap to build on to achieve the goals of precision cancer medicine.

## Author contributions

All authors listed have made a substantial, direct, and intellectual contribution to the work and approved it for publication.
